# INCREASED BETA2-ADRENERGIC RECEPTOR SIGNALING ENHANCES PROGRESSION OF HEPATOCELLULAR CARCINOMA

**DOI:** 10.1093/geroni/igz038.395

**Published:** 2019-11-08

**Authors:** Jason Pizzini, Hanzhou Wang, Chih-Ko Yeh, Amrita Kamat

**Affiliations:** 1 University of Texas Health Science Center, San Antonio, Texas, United States; 2 University of Texas Health at San Antonio, Texas, United States; 3 Geriatric Research, Education and Clinical Center (GRECC) South Texas Veterans Health Care System (STVHCS), San Antonio, Texas, United States

## Abstract

We investigated whether increased signaling by beta2-adrenergic receptors (β2-ARs), which mediate the action of catecholamines, enhances the progression of hepatocellular carcinoma (HCC). Mean age of patients with HCC, the most prolific form of liver cancer, has progressively increased over the last decade. Beta2-AR-mediated signaling in liver increases with age. We also observed increased β2-AR levels in liver tissues of patients with HCC compared to control subjects. We, therefore, hypothesized that increased β2-AR signaling enhances HCC progression while inhibition of β2-AR signaling by treatment with beta blockers suppresses its progression. To test this hypothesis, we used N-nitrosodiethylamine (DEN) to induce HCC in liver-specific β2-AR knockout (LKO) and control mice in the absence or presence of beta blocker propranolol. At the end of 25 weeks, we observed increased numbers of visible tumors, disarray of liver architecture, and mortality in DEN-induced control mice which was reduced by propranolol treatment. We also observed that DEN-treated LKO mice demonstrated reduced mortality, disarray of architecture, and phosphorylation of oncogene Src compared to DEN-treated control mice. Taken together, these results indicate that decreased β2-AR signaling because of a lack of receptors in the liver or inhibition of receptor action with propranolol reduces HCC progression. Studies are in progress to determine the β2-AR-mediated mechanisms involved in HCC progression. Our studies suggest that beta blocker propranolol, used to treat cardiovascular diseases, may be repurposed as a potential therapeutic option for treatment of HCC.

